# Artificial and Naturally Derived Phospholipidic Bilayers as Smart Coatings of Solid-State Nanoparticles: Current Works and Perspectives in Cancer Therapy

**DOI:** 10.3390/ijms232415815

**Published:** 2022-12-13

**Authors:** Nicolò Maria Percivalle, Marco Carofiglio, Marzia Conte, Giada Rosso, Alessandro Bentivogli, Giulia Mesiano, Veronica Vighetto, Valentina Cauda

**Affiliations:** Politecnico di Torino, Department of Applied Science and Technology, Corso Duca degli Abruzzi 24, 10129 Torino, Italy

**Keywords:** nanoparticles, liposomes, extracellular vesicles, lipidic shell, biomimicking coating, nanomedicine, supported lipid bilayer, cancer therapy, theranostics

## Abstract

Recent advances in nanomedicine toward cancer treatment have considered exploiting liposomes and extracellular vesicles as effective cargos to deliver therapeutic agents to tumor cells. Meanwhile, solid-state nanoparticles are continuing to attract interest for their great medical potential thanks to their countless properties and possible applications. However, possible drawbacks arising from the use of nanoparticles in nanomedicine, such as the nonspecific uptake of these materials in healthy organs, their aggregation in biological environments and their possible immunogenicity, must be taken into account. Considering these limitations and the intrinsic capability of phospholipidic bilayers to act as a biocompatible shield, their exploitation for effectively encasing solid-state nanoparticles seems a promising strategy to broaden the frontiers of cancer nanomedicine, also providing the possibility to engineer the lipid bilayers to further enhance the therapeutic potential of such nanotools. This work aims to give a comprehensive overview of the latest developments in the use of artificial liposomes and naturally derived extracellular vesicles for the coating of solid-state nanoparticles for cancer treatment, starting from in vitro works until the up-to-date advances and current limitations of these nanopharmaceutics in clinical applications, passing through in vivo and 3D cultures studies.

## 1. Introduction

Within the last decade, personalized medicine has emerged as an approach aiming to introduce effective, specific and safe solutions for the treatment of a disease, with customized features tailored to the individual patient. The use of nanomedicine, and in particular the application of nanosized materials and technologies to the personalized medicine approach, has raised a broad interest. In fact, an exponential increase in research has been witnessed in the biomedical and nanomedicine fields, bringing together expertise from biology, medicine, chemistry, physics, materials science and engineering disciplines in a common effort. Nanomaterials, and in particular nanoparticles (NPs), can be tailored to modulate their size, shape, surface charge, hydrophilic/hydrophobic nature, chemical composition and structure, as well as their surface reactivity and chemistry. Thus, there is an immense opportunity to play with them and try to tune the interaction of NPs with the biological environment. Recently, the major challenges in this field were unanimously recognized to be (i) obtaining biomimetic NPs, (ii) allowing smart and fully controllable functions, (iii) achieving a site-selective and timely targeting of the NPs to nullify any off-target administrations [1,2,3].

When formulating a synthetic nanomaterial, either organic or inorganic, with specific and tailored properties, it must be carefully considered that its characteristics can dramatically change once inserted into a biological environment. In fact, a nanoparticle does physically and chemically interact with the surrounding media and a new biological identity is defined, which will determine the physiological response of the body to the external entity. In particular, after the interaction with biological media components, nanoparticles can undergo aggregation, protein adsorption and material degradation, dissolution or speciation [4]. Thus, the role of biomimetism, once referred to as the biological identity of nanoparticles, is to combine synthetic and biological strategies enabling the mimicking of natural mechanisms, trying to overcome the hurdles associated with the delivery of nanoparticles in a biological environment, and preventing the formation of by-products and any undesired effects.

With the aim of developing biomimetic and high-performing imaging and therapeutic nanotools for personalized diagnosis and therapy, the strategy of incorporating synthetic NPs into lipid bilayer envelopes has been established. In fact, cells use lipid bilayers as natural envelopes, i.e., the cell membranes, thus constituting a natural barrier separating the cell interior from the extracellular space. Amphipatic lipids are macromolecules constituted by a hydrophilic polar head and a hydrophobic double tail. The combination of different head groups with a different number of acyl chains, possessing a peculiar length and saturation level, originates multiple classes of lipid molecules, such as phospholipids, glycolipids, sphingolipids, sterols, etc. [5]. The amphiphilic chemical structure commonly shared by all classes of lipids is responsible for the formation of their characteristic bilayer arrangement in aqueous and biological environments. In this arrangement, in fact, the lipids self-organize themselves in order to protect the hydrophobic tails from the surrounding media. Therefore, in view of these chemical, structural and biological functions, lipids are considered good candidates for the stabilization of NPs, providing a defensive and biocompatible barrier [6] and promoting a “safe” biological identity. In fact, several liposomes, i.e., spherical vesicles constituted by one or more lipid bilayers, are currently used as drug or mRNA nanocarriers in clinical applications [7]. More recently, lipids have been proposed as stabilizing agents to improve the colloidal and chemical stability of inorganic nanoparticles in the biological environment [8].

**Figure 1:**
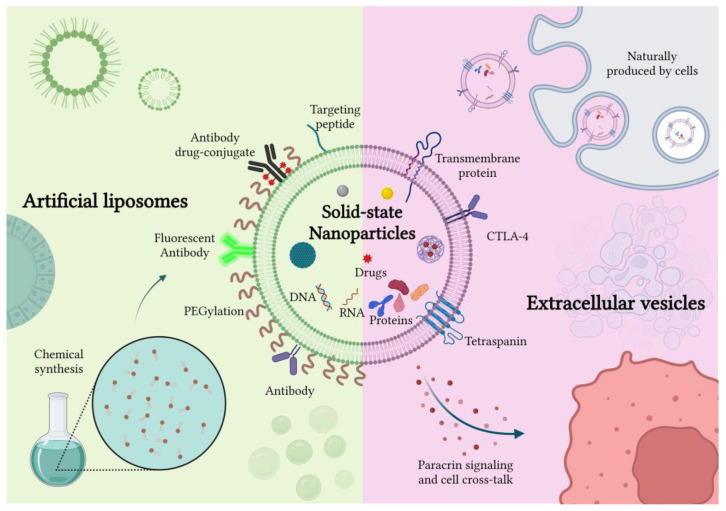
Schematic representation of artificial and naturally derived smart coatings for solid-state nanoparticles. Created with BioRender.com, accessed on 26 October 2022.

The present manuscript aims to review the lipid coating introduced on the surface of NPs: we propose a comprehensive overview of these newly conceived lipid-coated NPs, focusing on their use and application in vitro, in vivo, in 3D models and in the clinical panorama (Figure 1). The use of different types of phospholipids, applied either singularly or in mixtures, is the most widely proposed one in biomedical applications, especially to coat solid-state nanoparticles. Indeed, their polar heads, which expose different chemical groups, ensure their good interaction with several kinds of NPs [9]. Furthermore, they are also used in combination with other types of lipids, such as cholesterol or sphingomyelin [10].

Already around 30 years ago, naturally produced lipid bilayer vesicles were discovered: these are called extracellular vesicles (EVs) and are secreted by almost all types of eukaryotic cells and prokaryotic ones into the surrounding environment. EVs are more than lipid bilayer vesicles: they comprehend different types of cell-derived membranous lipids and proteins and contain cytosolic material and nuclear components as well. EVs are present in various biological fluids and are involved in different physiological and pathological processes. Recently, the International Society for Extracellular Vesicles (ISEV) proposed a new classification method based on the physico-chemical characteristics of EVs. For instance, EVs could be categorized as small (sEVs < 100 or 200 nm) or medium/large (m/lEVs > 200 nm) considering their dimension, or as low/middle/high dense EVs considering their density [11]. Thanks to their cellular origin and the biological role of intercellular messengers, EVs represent good candidates for the formulation of specific, nonimmunogenic and stable delivery vehicles for different therapeutic payloads. EVs possess natural stability in blood and other biological fluids and an intrinsic ability to cross different biological barriers, ascribable to their small size and particular structure, which ensure optimal extravasation capability and tissue penetration [12]. Moreover, their lipid bilayer allows the loading of either hydrophilic or hydrophobic compounds to be stable in the EVs’ core or membrane, respectively [13]. From this perspective, EVs can be assimilated to liposomes and proposed as nanocarriers for drugs, but more innovatively as carriers of inorganic nanoparticles. As liposomes, they could indeed provide a suitable defensive barrier to preserve the colloidal and chemical stability of different nanomaterials in the biological environment. However, in addition to this protective feature, EVs possess further characteristics with respect to synthetic liposomes, such as low immunogenicity, absence of toxicity and better biocompatibility [14]. In fact, from the perspective of personalized drug delivery systems, it was reported that EVs collected directly from patient blood or tissues (i.e., autologous EVs) could provide a well-tolerated and safe therapeutic option [15]. Several studies, in fact, reported the presence of particular receptors on the EVs’ surface which inhibit their interaction with the mononuclear phagocyte system (MPS), ensuring a lower clearance and a better biodistribution [16]. In addition to that, some in vitro studies indicate that EVs could also possess intrinsic tropism and could be selectively distributed to particular organs and tissues, thanks to their peculiar molecular composition, which is precisely controlled by their cellular source [17].

Considering these promising features, this review also discusses the most relevant advances of solid-state nanoparticles incorporated into EVs for in vitro and in vivo applications and the related treatment modality.

In addition to the most recent results, hybrid envelopes constituted by a fusion of EVs and liposomes, as well as fully synthetic EV-biomimetics, are reviewed here. As will be shown below, the field is currently emerging, leading to exciting applications of personalized nanomedicines, not only against cancer but also involving other, different diseases.

Finally, this work briefly introduces the main drawbacks and limitations currently preventing the use of most nanomedicines and nanopharmaceutics (considered in their most general terms) in clinical trials, highlighting the main challenges related to their application and shedding light on future innovative treatments.

## 2. In Vitro Studies

### 2.1. Artificial Liposomes-Coated NPs

The introduction of liposomes as drug delivery systems (DDSs) during the 1960s was one of the most important breakthroughs in nanomedicine [18]. Thanks to their unique properties as nanocarriers, their application enabled an enhancement of the therapeutic indexes of chemotherapy drugs-based treatments [19,20] and resulted in the approval of some successful liposomal formulations for cancer therapy by the Food and Drug Administration (FDA) [21,22,23].

Over the last decades, the variety of available liposomes has remarkably increased and evolved from conventional vesicles to smart liposomes exhibiting surface modifications, such as PEGylation, intrinsic thermo- or pH-sensibility, functionalization with peptides and antibodies, to better target the tissues of interest [24]. Accordingly, many techniques for their fabrication and characterization have been implemented as well, allowing the very precise tuning of their surface properties and their composition [25,26].

Since one of the most challenging issues related to cancer therapy is the complexity and heterogeneity of tumor biology, and consequently the peculiar nano–bio interaction taking place between tumors and nanomedicine tools [3,27,28], a precise control of their chemical and physical properties must be taken into serious consideration [29,30]. As far as inorganic nanoparticles are concerned, regardless of their composition, a major drawback of their use is related to their aggregation in biological fluids and, thus, to their short circulation life, which hinders their effective localization into the target organs and triggers a rapid immune response [31,32]. An effective shielding with phospholipids able to mimic cellular membranes could, therefore, be the most straightforward strategy to impart them with stealth properties and to enhance their delivery by means of passive transport, such as the enhanced permeation and retention (EPR) effect. Moreover, by introducing targeting strategies in the form of surface functionalizations, an active transport could be exploited as well, while also generally improving the toxicity profile of the lipid-coated nanoparticles with respect to pristine ones [33,34].

In the context of lipid coatings for inorganic nanoparticles, two different arrangements can be employed to introduce lipid molecules on the NP surface: supported lipid bilayers (SLB), namely when lipids adopt the typical lipid bilayer by exposing their hydrophilic heads toward the NP surface (by means of electrostatic and van der Waals interactions), forming an inner and an outer leaflet with the same type of molecules, and hybrid lipid bilayers (HLB), in which the outer leaflet composition is different from the inner one [6]. Consequently, the fabrication procedures of SLBs and HLBs follow substantially different steps, and some related applications are hereafter reported. SLBs are mainly applied to metal or metal oxide surfaces, provided that they expose a hydrophilic surface, such as silica [26,35,36,37,38,39,40], gold [41,42,43] and silver NPs [44,45].

If the synthesis process of the core NPs requires the presence of a capping agent, instead, this pre-existing leaflet of molecules can be exploited as a platform for the lipid coating, leading to HLBs. Typical capping agents are amine, carboxylic acids, thiols or, in the case of lipids, oleic acid as well; they are usually employed to stabilize super paramagnetic iron oxide nanoparticles (SPIONs), quantum dots (QDs) and metal oxide NPs [46,47].

It is, therefore, clear, based on what has been mentioned so far, that the nature and the synthetic route of solid-state nanoparticles can drive the selection of a lipid bilayer type among SLBs or HLBs. Furthermore, the solid-state nanoparticle can fulfil a specific function, enabling the whole core-shell system to be used for drug or gene delivery, bio-imaging or stimuli-responsive actions.

As a prominent example in this sense, mesoporous silica nanomaterials are typically proposed for drug incorporation, thanks to their high surface area and pore volume. Therefore, the ability of lipid-coated silica NPs to incorporate huge amounts of payloads in their mesoporous core is one of the key aspects of their nanomedicine use. The presence of the lipid coating, in fact, prevents the entrapped drugs from unwanted leakage while enhancing their biocompatibility and avoiding their aggregation in a biological medium. These key points were highlighted by a study from Durfee and colleagues, who developed a protocol consisting of the lipid vesicle fusion onto cargo-loaded monosized mesoporous silica nanoparticles (mMSNs) followed by antibody conjugation to form targeted mMSN-supported lipid bilayers, called Protocells. After a thorough physico-chemical characterization, they successfully tested them in vitro, proving their biocompatibility, targeting abilities and enhanced drug delivery due to the presence of the lipid bilayer (made of 1,2-distearoyl-sn-glycero-3-phosphocholine (DSPC), 1,2-distearoyl-sn-glycero-3-phosphoethanolamine-N-[methoxy(polyethylene glycol)-2000] (DSPE-PEG_2000_), 1,2-distearoyl-sn-glycero-3-phosphoethanolamine-N-[amino(polyethylene glycol)-2000] (ammonium salt) (DSPE-PEG_2000_ Amine), and cholesterol, following precise molar ratio) [48], and successfully ex ovo and in vivo as well. Another work had previously reported the implementation of a supported lipid bilayer on colloidal mesoporous silica nanoparticles by means of the solvent exchange technique, obtaining colloidally stable hybrid systems with a high potential for drug delivery [49].

Among the many and diverse nanoparticle applications, the incorporation of paramagnetic agents such as iron oxide into thermosensitive liposomes to impart them with magnetic field sensitivity was one of the strategies implemented in order to better localize the nanoconstructs in tumor sites [50,51]. Another approach to improve the traceability of drug carriers consisted of the use of liposome-coated near-infrared persistent luminescence NPs loaded with paclitaxel, whose application resulted in a high drug loading and excellent imaging abilities both in vitro and in vivo [52].

In the last decades, zinc oxide nanoparticles have shown great potential in nanomedicine and have been employed in cancer therapy thanks to their selectivity in inducing cytotoxic effects on tumor cells [53]. Since a salient point underlying their application is possessing colloidal stability to avoid unwanted aggregation and toxicity, many works have focused on the implementation of coatings intended to provide them with a higher biocompatibility and biostability. A study by Zeng et al. reported the use of a lipid coating consisting of an inner leaflet of 1,2-dioleoyl-sn-glycero-3-phosphate (sodium salt) (DOPA) and an outer leaflet of a mixture of 1,2-dioleoyl-sn-glycero-3-phosphocholine (DOPC), cholesterol and DSPE-PEG_2000_ on zinc oxide nanoparticles, obtaining core-shell structures containing 6-mercaptopurine (6-MP). In vitro experiments proved their strong preferential ability to kill cancerous cells, possibly due to the generation of radical oxygen species (ROS), and the enhanced effect of 6-MP when loaded into the nanoparticles, probably due to an improved internalization through endocytosis and a rapid release to the cytoplasm [54]. Another application of lipid-coated zinc oxide nanoparticles was reported by Dumontel and colleagues, who proved the enhanced colloidal stability of such nanoconstructs when immersed in simulated human plasma and in a cell culture medium with respect to pristine nanoparticles. Moreover, they hypothesized that the presence of a lipidic shell could prevent a premature degradation of zinc oxide into toxic by-products, thus increasing its cytotoxicity, and evidenced a higher rate of internalization compared to uncoated zinc oxide samples [8].

Lipid-polymeric NPs, such as lipid-poly(lactic-co-glycolic acid) (PLGA) ones, have been extensively studied over the last decades, and a comprehensive review published in 2015 reported their main formulation methods and theranostic approaches [55]. A recent study employed them as drug carriers for Paclitaxel, with the aim of combining the structural integrity of the PLGA core and the customizable nature of the lipidic shell by varying the surfactant in the object, for both in vitro and in vivo applications [56]. A few years before, engineered lipid layers on PLGA nanoparticles had already been applied in vitro to assess the influence of some of their components, such as the amount of cholesterol in the lipidic formulation, in terms of the cellular uptake and cargo release [57]. Lipid-enveloped pH-responsive polymer NPs were also studied as mRNA delivery systems, both in vitro and in vivo, in a proof-of-concept study by Su et al., showing great potential as vaccine vehicles [58]. The superiority of lipid-polymeric NPs over liposomes as delivery systems was echoed by a subsequent work by Ayad and co-workers, who employed a solid core of poly(lactic) acid (PLA) to guarantee a higher cargo retention, in that case mRNA, and to provide the resulting carrier with a higher versatility toward different nucleic acid payloads [59]. Another study reported that the presence of lipids on top of pH-responsive drug delivery platforms consisting of polyacrylic acid/calcium phosphate not only did not affect their pH-responsive mechanism of action at all, but also enhanced their cellular uptake in vitro [60].

**Figure 2:**
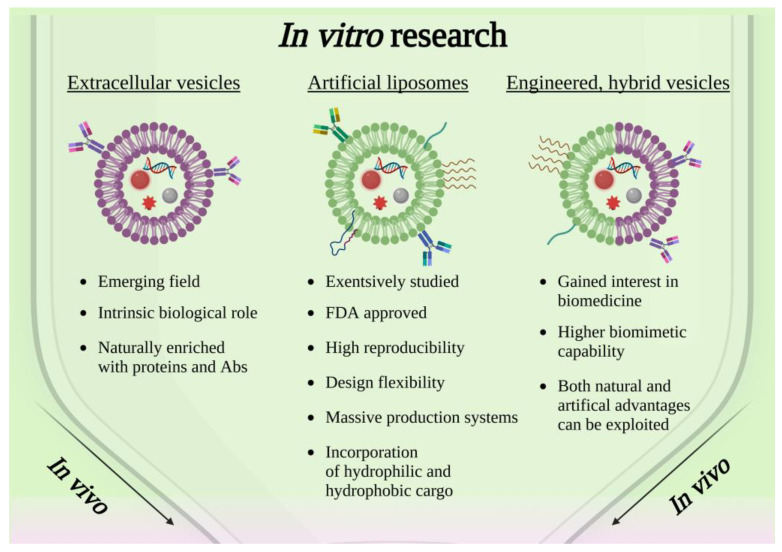
Scheme showing the current panorama of in vitro research involving solid-state nanoparticles enclosed in lipidic-based shell, extracellular vesicles or engineered hybrid vesicles. Created with BioRender.com, accessed on 26 October 2022.

Taken together, these works confirmed the efficacy of a lipid-coating strategy on different types of nanoparticles and the near absence of any negative influences on the material at the core of the resulting nanoconstructs, which is a crucial aspect to consider. Moreover, since the use of liposomes is supported by the extensive literature regarding their stand-alone role as drug delivery systems [61,62], and they are typically composed of FDA-approved chemical species, their employment as a coating arouses few biocompatibility concerns. Some of the above-mentioned advantages are resumed in the scheme shown in Figure 2.

Nonetheless, the high reproducibility of artificial systems based on commercially available lipids is counterbalanced by their struggle to naturally promote cellular crosstalks or paracrine signaling in the targeted cells. For this reason, it is of the utmost importance to carefully study and tune the composition of liposomes as well as their possible interaction with membrane proteins, by means of a careful observation of the biological membranes [63]. Therefore, a growing body of the literature (Table 1) has investigated the employment of cell-derived extracellular vesicles (EVs), whose innate role in cell communication and membrane natural composition suggests that their use as a coating on solid nanoparticles could be a major step forward with respect to liposomes.

### 2.2. Cell-Derived Extracellular Vesicles Coated NPs

As has been reviewed so far, the exploitation of phospholipidic bilayers to mimic cellular membranes is a powerful method to enhance the biostability, biocompatibility and biodistribution of organic and inorganic NPs, whose employment in nanomedicine would be otherwise very limited. However, the use of an easily recognizable-as-safe entity to the organism to conceal therapeutic or imaging moieties represents a potent as well as tricky engineering problem. Fully functional cargos are already produced by cells themselves and are exploited by the living organism to deliver information, nutrients or, in general, biomolecules [64,65]. Extracellular vesicles represent the clearest example of that. Based on their size and genesis, EVs play different roles in cell biology and could be employed differently for the fabrication of stealth nanoparticles. A first classification can be performed by considering the size of such EVs, between small EVs (sEVs, <100 or 200 nm) and medium/large EVs (m/lEVs, >200 nm) [11]. Extracellular vesicles are particularly suitable for the incorporation of NPs, because they can be potentially isolated from all body fluids or commercial cell growth mediums once cells have been cultured in it. The control on their composition is less strong with respect to the ones obtained from commercially available lipids, and their extraction and conservation more difficult. However, the complex equilibria which are present in naturally produced membranes ensure a general cytocompatibility, and an enhanced specificity toward the cells from which they are derived, paving the way for powerful theranostic hybrid inorganic–organic nanodevices for cancer therapy.

The intuitive but difficult task of using EVs as drug nanocarriers has been investigated in the literature [66,67]. However, most studies are still focused on their characterization, isolation and conservation rather than on their exploitation as a tool for nanomedicine. When considering the coating of solid nanoparticles with EVs, a further engineering problem rises. As a matter of fact, the coupling of EVs with solid nanoparticles is far from being a straightforward process. The possibilities comprehend (i) the coupling by means of the coulombic interaction occurring between oppositely charged entities, (ii) extrusion through nanosized pore membranes, (iii) freeze–thaw or sonication or electroporation processes aimed at the temporary disruption of EVs and their redialing in a medium rich in NPs, and (iv) the coincubation of NPs with cells and subsequent extraction of NP-loaded EVs. Despite the technical difficulties in reaching these ambitious tasks, some preliminary in vitro works (in which EVs were employed to ferry solid NPs) have already been published, as detailed below.

A clear example of solid nanoparticles encapsulated into EVs to increase their biostability and biocompatibility can be found in the work of Srivastava et al. [68]. They showed the possibility of embedding gold nanoparticles aimed at doxorubicin delivery into exosomes. Doxorubicin was conjugated with gold NPs through a pH-sensitive bond which can be cleaved in acidic conditions. The embedding of EVs was reached by a simple electrostatic attraction between the positive NPs and the negative phospholipidic membrane. Therefore, such EV-incorporated nanoparticles gathered an enhanced biocompatibility which also resulted in a selectivity of the toxicity mechanism toward tumoral cells.

In another research paper, hollow gold nanoparticles were included in human placental mesenchymal stem cells (MCS)-derived exosomes. In this work, exosome-coated Au NPs were obtained after the co-incubation of the NPs with the MCS cells and the successful isolation of exosomes by ultracentrifugation. It was demonstrated that EV-covered NPs were selectively transferred to the cell type of origin, proving the potentialities of the method for the treatment of specific diseases such as cancer [68].

Another work involving gold-iron oxide nanoparticles (GIONs) sees the coverage of the nanoparticles with EVs by extrusion through a 200 nm pore membrane. In this case, the aim was to obtain a multimodal theranostic device for anticancer therapy. Tumor-derived EVs acted as carriers of both microRNA and GION theranostic nanoparticles, which can act as a photosensitizer for photothermal therapy and as a contrast agent for magnetic resonance imaging [69].

Recently, metal-organic frameworks (MOFs) were also considered as carriers for anticancer drug delivery. However, these MOF NPs require sealing mechanisms to avoid drug leakages during both administration and blood circulation before reaching their target. Exosomes, and more generally EVs, were proposed as shields for these systems preventing premature drug release [70]. Cheng et al. proposed the exploitation of MDA-MB-231 tumor cell-derived EVs to envelope an MOF nanoparticle loaded with proteins. Extrusion and sonication were the processes exploited for the coverage of the protein-loaded MOF NPs with EVs. The results showed a reduced opsonization and phagocytosis by a murine macrophage-like cell line and an increased internalization for cells from which EVs were derived, demonstrating the potential of these hybrid nanoconstructs in anticancer therapy [71].

Interestingly, zinc oxide nanoparticles were embedded into cancer cell-derived EVs from the cervical adenocarcinoma cell line [72] and in B-lymphocytes-derived EVs [73]. In both cases, ZnO NPs were combined with EVs to prevent the dissolution into Zn^2+^ ions and the consequent ZnO toxicity. The incorporation was carried out by either a simple co-incubation method when combining ZnO to cancer-derived EVs or by means of a freeze–thaw method when using the lymphocyte-derived ones. Not only did the EVs increase the internalization of the nanoconstruct inside the cancerous cells, but they also allowed the release of the EVs’ cargo only inside the recipient cancer cells, acting as potent nanodrugs [72].

Another metal oxide that has been efficiently embedded into exosomes is iron oxide. In the work of Piffoux et al., iron oxide nanoparticles, together with a photosensitizing agent (Foscan), were embedded into EVs with the aim of producing a theranostic anticancer nanoconstruct with specific magnetic and light responsiveness. In fact, these features could be exploited to magnetically manipulate exosomes and to detect their position by means of magnetic resonance imaging or fluorescence detection [74].

In another study, a novel radiosensitizer with high selectivity was also developed thanks to its coating with tumor-derived exosomes [75]. Manganese carbonyl was loaded into exosomes to target the tumor and to synergically act with radiotherapy in vitro against cancer cells.

Some works also report polymeric nanoparticles that were covered with EVs. Recently, poly(2-ethyl-2ozazoline)-poly(D,L-lactide) (PEOz-PLA) was also loaded with doxorubicin and coated with urinary exosomes. In this work, the idea was to exploit a body fluid that can be picked up by the patient without invasive processes to obtain tumor-targeting exosomes. In vitro results showed an enhanced rate of apoptosis when employing these nanoconstructs, which was attributed to the increased targeting ability of cancer patient-derived exosomes [76].

In conclusion, as also reported in Figure 2, the coating of solid nanoparticles with extracellular vesicles is a promising field in cancer therapy which is, however, in its early stages. Still, some works proposing EVs as a cargo for drug delivery-aimed nanoparticles are present in the literature and very few works propose EVs shielding intrinsically active theranostic nanoparticles. In general, EVs produced for this purpose are derived from tumoral cells and present, as a main advantage, the selectivity toward the cell line of origin, ascribable to the complex protein system that characterizes them.

### 2.3. Engineered, Hybrid and Artificial Extracellular Vesicles 

As reported in the previous sections, both liposomes and extracellular vesicles have gained growing therapeutic interest in recent years due to their properties, which make them extremely suitable for nanomedicine research and applications. EVs have been extensively studied as possible nanocarriers to exploit their natural biological role for therapeutic purposes [77,78]. However, there are still some potential drawbacks and unknown aspects that can limit their medical applicability, such as difficulties in EV extraction (with particular focus on the process yield) [79], characterization, mass production [80] and their rapid clearance from the blood when EVs are systemically administered [81]. To overcome these difficulties, different approaches have been explored, starting from the extracellular vesicle surface modification to obtain engineered EVs, up to the development of hybrid EVs achieved through the fusion of their membrane with liposomes to combine the unique features that these two organic structures provide. Finally, there is growing interest in the development of artificial EVs, characterized by a lipidic bilayer composed of artificial lipids enriched with specific proteins in order to mimic the biological features of natural extracellular vesicles. While engineered EVs have been studied as potential biocompatible and biomimetic shells for NP coating, the literature is still quite poor in terms of examples regarding hybrid and artificial EVs for NP encapsulation. However, these two approaches are at the heart of nanomedicine research, especially as effective drug delivery systems. A near future in which hybrid and artificial EVs are exploited as an NP coating (with the possible addition of an anticancer drug to enhance the therapeutic potential of the nanoconstructs) seems a viable and promising option.

As mentioned above, a first possible strategy to improve the delivery efficacy of natural EVs is to modify and modulate the components of the natural vesicles, thus creating “tailor made” vesicles, adapting them to a specific purpose [82]. For example, EVs can be decorated with additional targeting or functional moieties, such as peptides [83], small proteins [84] or fluorescent proteins [85]. Such artificially modified EVs are called “engineered EVs” and can be obtained mainly through two approaches: the genetic engineering of the parental cells from which the EVs are extracted [86], and the direct modification of the natural occurring EVs, once isolated [87]. Regarding the direct method, a very frequent approach is PEGylation, which was proven effective in extending the half-life of the nanovesicles and improving their tissue accumulation [88].

EV engineering has, therefore, the potential to enhance the stability and circulation time, improve specific targeting and boost up intracellular uptake in the target recipient cells. However, the main drawback of this approach is the possibility of altering the orientation of natural surface proteins, thus compromising their related biological function [89].

In the context of nanoparticles coated with engineered EVs with anticancer scopes, the scenario is still narrow and great leeway is left to researchers. In general, nanoparticles are typically inorganic and exploited to obtain a stimuli-responsive therapy, to boost the cytotoxicity of another loaded therapeutic agent.

One of the few examples found in the literature is provided by Dumontel et al. [73], who, interestingly, coated therapeutical and stimuli-responsive zinc oxide nanocrystals (ZnO NCs) with engineered B-cell-derived EVs, obtaining the so-called “TrojanNanoHorse” (TNH) nanoconstruct against Burkitt’s lymphoma cells. In particular, ZnO NCs were embedded in the engineered vesicles through a freeze–thaw method, conferring to the ZnO NCs’ stealth properties and improving their colloidal stability. EVs were modified after the isolation and further decorated with an anti-CD20 monoclonal antibody, in order to achieve specific targeting toward lymphoid cancer. ZnO NCs possess intrinsic cytotoxicity and, in this case, were activated by an external stimulus, i.e., acoustic shock waves, demonstrating the efficacy and on-demand synergistic cytotoxicity of these TNH nanoconstructs.

Another great example of engineered EV-embedded nanoparticles was reported by Jia et al. [90]. In this work, curcumin-loaded superparamagnetic iron oxide nanoparticles (SPIONs) were loaded into exosomes (Exos) isolated from the Raw264.7 macrophage cell line. In this case, the surface of the Exos was conjugated by means of click chemistry with neuropilin-1-targeted peptide (RGERPPR, RGE), in order to obtain specificity toward glioma cells. The resulting nanoconstruct was demonstrated to be suitable for imaging and SPION-mediated magnetic flow hyperthermia therapy, which, in addition, showed a strong synergistic activity with curcumin against the human glioma cell line U251.

In a recent work, Li et al. [91] exploited macrophage-derived exosomes to efficiently coat PLGA NPs, previously synthesized through a nanoprecipitation method and loaded with doxorubicin, to perform chemotherapy against triple-negative breast cancer (TNBC). The coating process was carried out by exploiting the coextrusion technique. To further improve the targeting capability of the nanoconstructs, the exosomes’ surface was engineered with the addition of a binding peptide with a high affinity to the mesenchymal-epithelial transition factor (c-Met), which is overexpressed in TNBC cells. The in vitro tests showed that the doxorubicin cellular uptake, as well as its cytotoxicity, increased when the drug was delivered through the nanotool compared to its administration as a free drug.

A possible alternative approach which reached the in vivo step was proposed by Kwon et al. [92], consisting of exosome-based nanoconstructs bound to magnetic nanoparticles (MNP). Notably, in this case, the nanoparticles were not embedded in the core of the engineered EVs but exposed on the vesicles’ membrane. The MNPs were loaded with doxorubicin and bound to HT-29 colorectal cancer cell-derived exosomes thanks to the functionalization of the MNPs with epithelial cell-adhesion molecules. The targeting ability of exosomes was, in this case, enhanced through conjugation with folic acid; the engineered nanoconstructs were tested on HT-29 cells, exploiting the combined action of doxorubicin and an alternate magnetic field-driven hyperthermia, proving the presence of a synergistic effect of the two treatments.

The development of a hybrid lipidic shell composed of EVs (eventually already engineered) and liposomes is an innovative approach that allows a benefit from the advantages offered by the biological properties of natural extracellular vesicles, while enhancing the therapeutic capabilities of the obtained nanoconstructs by finely tuning the composition of the synthetic counterpart [79]. This procedure guarantees EVs’ enhanced colloidal stability and half-life in blood, and enables the increase in the cargo encapsulation efficiency [93].

Many recent works focused their attention on such hybrid Evs regarding the development of DDSs. Piffoux et al. [94] tested different combinations of extracellular vesicles and liposomes by exploiting a polyethylene glycol (PEG)-mediated fusion to obtain biosynthetic hybrid vectors. The study results highlighted the possibility of tuning the properties of the hybrid EV, while also showing the enhanced drug-loading capability of such nanoconstructs with respect to synthetic liposomes.

Rayamajhi et al. [95] exploited a film hydration followed by a membrane extrusion method to synthesize hybrid exosomes (HE) composed of immune cell-derived small extracellular vesicles (sEVs) and synthetic liposomes. In vitro tests on cancer and normal cell lines showed increased toxicity toward tumor cells, making these HEs an interesting platform for the development of DDSs.

In another work, Lv et al. [96] tried to overcome the drawbacks of hyperthermic intraperitoneal chemotherapy (HIPEC) for the treatment of metastatic peritoneal carcinoma (mPC) by designing a hybrid EV fusing fibroblast-derived exosomes with thermosensitive liposomes. Furthermore, granulocyte-macrophage colony-stimulating factor (GM-CSF) and docetaxel were co-loaded into the obtained nanoconstructs. The study demonstrated that their administration increased the therapeutic efficacy of HIPEC, while showing great potential for the systemic co-delivery of chemotherapeutic agents.

Li et al. [97] addressed cisplatin resistance during the treatment of ovarian cancer (OC). To overcome this problem, the authors tried to administer microRNA-497 (miR497), which may inhibit a specific pathway involved in chemotherapy resistance, and triptolide (TP), which is able to effectively kill cisplatin-resistant cell lines by co-loading them into a hybrid EV composed of tumor-derived exosomes and cRGD-modified liposomes, with encouraging in vitro results.

Given the promising results of the above-mentioned works, which just consider the combination of pristine EVs with empty or drug/gene-loaded liposomes, it may be assumed that nanomedicine research can expand its frontiers toward the development of therapeutic nanoconstructs in which hybrid EVs are used to encase solid-state NPs. A recently published study performed by Barui et al. [98] fits into this context. In this paper, the authors developed an innovative hybrid lipidic shell composed of B-lymphocytes-derived EVs and artificial lipids to encapsulate Gadolinium-doped, gemcitabine-loaded ZnO NPs for the treatment of pancreatic cancer. The nanoconstructs, obtained through the freeze–thaw technique, showed great colloidal stability in a biological environment and enhanced magnetic behavior thanks to the presence of the doped NPs, which may be exploited for diagnostic purposes. In vitro tests demonstrated promising results in terms of cellular uptake, and a greatly enhanced gemcitabine cytotoxicity toward cancer cells when the drug was administered through the nanoconstructs. A review of the in vitro application of engineered extracellular vesiscles is reported in the scheme of Figure 2.

Natural EVs possess intrinsic powerful and desirable characteristic for a nanosized therapeutical DDS, such as high intracellular efficiency, precise targeting capability, circulation stability and the ability to overcome biological barriers, but not all the components of natural EVs are responsible for these biological and functional behaviors [99,100]. Based on this idea, a novel alternative approach is emerging, with the scope of building biomimetic, EV-inspired, synthetic nanodelivery systems, referred to here as “fully artificial EVs” (FAEVs). This name stresses out the fact that the concept is to artificially build them in a bottom-to-up approach, starting from well-characterized biomolecules (principally phospholipids and proteins) and employing only the key elements, to obtain an EV-resembling nanotool, with the desired functionalities. What is notable is that this strategy allows the possibility to bypass some of the obstacles related to natural EVs, such as the difficulty of EV extraction and purification methods and the lack of standardized procedures; moreover, it paves the way for a more controlled, safer product with a higher pharmaceutical acceptability [79].

In principle, FAEVs are synthetic lipid bilayers which are then functionalized with surface proteins, but, in this case, the composition is inspired by a natural one, according to the aim of the DDS. Therefore, for the nature of this approach, the complexity of the synthetic system will always be inferior to the biological counterpart, which is both a strength, since the system counts less variables and is easier to manipulate, but also the major drawback, since a necessary (and difficult) simplification is implied in the identification of the key components of the natural elements. The risk is that simplification could be too gross to reach the same performances in terms of the delivery efficacy of natural EVs.

This type of approach is, in general, very challenging, because it requires a detailed understanding of the role of many of the elements composing natural vesicles and the ability to combine them. For these reasons, research in this field is still in its infancy and examples in the literature are very rare and focused on a preliminary characterization of the FAEVs [101,102].

To the best of our knowledge, no study on solid-state nanoparticle-loaded FAEVs has been reported so far. However, it must be said that, thanks to the great similarity of FAEVs and EVs, and even more with liposomes, all the nanoparticle encapsulation techniques in EVs and liposomes could be easily transferred and adapted to FAEVs.

The first proof of concept of the potentiality of FAEVs as an effective alternative to EVs in the context of anticancer research was provided by Vázquez-Ríos and colleagues [103], who managed to develop exosome-mimetic nanosystems (EMNs), starting from the lipidomic studies of tumoral exosomes. The researchers here demonstrated that the artificial simplified version of EVs not only resembled its natural counterpart in terms of structural characteristics, but also maintained the same functional features of interest: the nanovehicolation of biological active moieties (EMNs were successfully loaded with miRNAs) and targeting specificity (EMNs were functionalized with integrin α6β4, in order to achieve lung organotropism).

In fact, the study by Vázquez-Ríos proved the possibility to obtain, even with a substantial simplification of their complexity, FAEVs with great similarity to natural reference EVs. EMNs were proven to be reproducible and easier to manufacture, prospecting a future in which safe multifunctional FAEVs could be designed, ad hoc tailored and large-scale produced to respond to specific therapeutical demands.

**Table 1:** In vitro studies on solid-state nanoparticles enclosed in lipidic-based shell, extracellular vesicles or engineered hybrid vesicles. Green background color refers to artificial liposomes, pink refers to extracellular vesicles and blue to engineered, hybrid and artificial extracellular vesicles.

NPs	Coverage-Liposome	Cargo	Functionalization	Purpose
Mesoporous silica	Liposomes (DSPC, DSPE-PEG_2000_, DSPE-PEG_2000_ Amine and cholesterol)	Gemcitabine	Anti-EGFR antibody	Drug delivery [48]
Persistent luminescence NPs	Liposomes (DSPC, DPPC, DOPC)	Paclitaxel	-	Drug delivery and imaging [50,51]
ZnO	Liposomes (DOPA DSPE-PEG200, DOPC)	6-MP	-	Drug delivery [54]
MOF NPs	Liposomes (DOPC)	-	-	MIL-100(Fe) MOF NPs dissolve intracellularly inducing pyroptosis [104]
PLGA	Liposomes (stearyl amine and soya lecithin)	Paclitaxel	Various stabilizers (PVA, F-68, TPGS, HAS)	Drug delivery [56]
PLA	Liposomes (DSPC/DOTAP)	microRNA	LAH4-L1 peptide	Gene therapy [59]
**NPs**	**Coverage-EV**	**Cargo**	**Functionalization**	**Purpose**
Au NPs	Tumor derived EVs	Doxorubicin	-	Drug delivery [68]
Hollow Au NPs	MSCs derived exosomes	-	-	Optical hyperthermia [105]
Au-iron oxide NPs	Tumor derived EVs	microRNA	-	Photodynamic, MRI and gene therapy [69]
MOF NPs	Tumor derived EVs	Calcein (proof of concept)	-	Drug delivery [70]
ZnO NPs	Cancer cells derived EVs	-	-	ZnO acting as a nanodrug inducing cytotoxicity to cancer cells [72]
ZnO NPs	Blood cells derived EVs	-	Anti-CD20 monoclonal antibody	ZnO assisted cancer therapy [73]
Iron oxide NPs	Endothelial cells derived exosomes	Photosensitizing agent (Foscan)	-	Photodynamic therapy and magnetic manipulation [74]
MnCO	Tumor derived exosomes	-	-	Radiotherapy [75]
PEOz-PLA NPs	Urinary exosomes	Doxorubicin	-	Drug delivery [76]
**NPs**	**Coverage-Hybrid**	**Cargo**	**Functionalization**	**Purpose**
Zinc oxide NPs	Blood cells derived EV-liposomes (DOPC)	Gemcitabine	CKAAKN peptide	Targeted and improved pancreatic cancer treatment [98]

## 3. In Vivo Studies

### 3.1. Artificial Liposome-Coated NPs for In Vivo Applications

Further advancements toward the translation of nanomedicine research into clinically approved therapy involve in vivo studies on the biodistribution, clearance and therapeutic efficacy of lipid-coated nanoconstructs. Even though the first drug-loaded liposomes were clinically approved in 1995 [106] and are nowadays well known in the healthcare sector as drug delivery systems, such as Doxil^®^, Ambisome^®^, or DepoDur™, the study of liposomes loaded with solid-state nanoparticles in vivo is still in its infancy. Most of the in vivo analysis reports the biodistribution of the nanoconstructs or the targeting efficiency, but just a few of them also tested the proposed therapeutic efficacy.

The state of the art of lipid-coated nanoparticles in biomedicine shows that a considerable assortment of nanomaterials with different natures can be enveloped in liposomes, from imaging to therapeutic applications.

**Figure 3:**
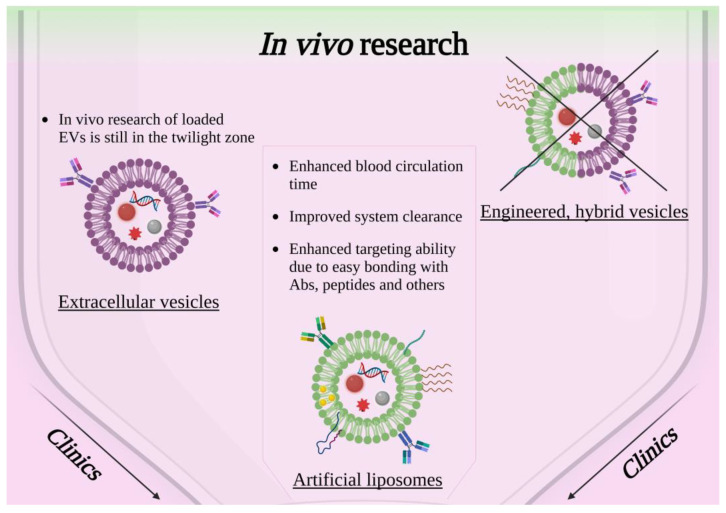
Scheme showing the current panorama of in vivo research involving solid-state nanoparticles enclosed in lipidic-based shell, extracellular vesicles or engineered hybrid vesicles. Created with BioRender.com, accessed on 26 October 2022.

Gold nanoparticles are among the most investigated liposome cargos in biomedicine, possibly for their adaptability and biocompatibility [107]. Localized resonant plasmons are generated on the surface of nanosized gold particles [108], and this phenomenon is often exploited for photothermal approaches in cancer therapy. Jeon et al. investigated the effect produced in vivo of a complex Au Liposome theranostic nanoconstruct [109]. They were able to synthesize liposomes, which were initially covered with gold NPs and successfully enveloped in other liposomes, creating a more stable and composite structure. The results demonstrated an excellent passive targeting ability, long circulation time and validated the efficacy of the photothermal treatment in vivo. To obtain a theranostic nanotool, this research group was able to functionalize their construct by radiolabeling it with ^64^Cu, and so they were able to acquire positron emission tomography (PET) images in vivo. Gold nanoparticle-aided photothermal therapy was also exploited in the study of Prasad et al., in which the cargo was composed of gold NPs and emissive graphene quantum dots, all enclosed in liposomes and further functionalized with folic acid ligands [110]. Liposome coverage ensured biocompatibility, hemocompatibility and easy degradation in vivo, as shown in Figure 3, the double cargos guaranteed a dual imaging modality and the targeting folic acid revealed a strong tumor-binding capability. The nanosystem, when exposed to near-infrared light, was demonstrated to generate photothermal heat and reactive oxygen species (ROS), also acting as a therapeutic agent. A reduction in the tumor in vivo was obtained, thanks also to the chemotherapeutic drugs loaded on to the NP surface. Despite most nanoconstructs being constituted by Au NPs covered with a lipidic shell, some other exotic solutions can be found in the literature. For example, Rengan et at. proposed a liposome-based system functionalized with very small gold nanoparticles (5–8 nm), bound to the liposome surfaces [111]. Tests performed on small animal models demonstrated the therapeutic potential of these constructs thanks to the photothermal therapy actuated through Au NPs, with an improved system clearance thanks to the biodegradable nature of the vehicles, i.e., the liposomes. Similarly, Zhu et al. studied a thermosensitive liposome enclosing ruthenium(II) polypyridyl complexes as an antitumor therapy in vivo [112]. Their strategy was to involve nanorod-shaped gold particles as the targeting agents of the liposome surface to allow the accurate release of ruthenium at the tumor site, avoiding the poor solubility and biocompatibility problems of the sole ruthenium.

Lipid-coated nanoparticles were mainly considered as a possible alternative to the clinical contrast agents used for imaging, due to the improvements that can be achieved in terms of biocompatibility, pharmacokinetics, biodistribution and targeting. To ensure the progress of the field, magnetic and nonmagnetic nanoparticles covered with lipidic shells were studied in vivo. On account of their magnetic nature, hydrophilic magnetite nanoparticles were enveloped in neutral liposomes by German et al. [113]. They demonstrated the in vivo safety of the designed magnetic nanoconstruct and evaluated its contrasting properties during magnetic resonance imaging (MRI) performed on transplanted renal carcinoma cells in rats. On the other hand, Chen et al. proposed superparamagnetic iron oxide nanoparticles which exhibited T1 magnetic resonance contrast enhancement, encased in liposome with targeting capabilities due to the conjugation with tumor-penetrating peptide (RGERPPR) [114]. This work validated the satisfactory MRI imaging capability of the nanoconstruct avoiding major cytotoxic effects and demonstrated the increased targeting aptitude toward glioma cells with respect to the not targeted lipid-coated NPs and their enhanced permeability and retention effect.

In vivo imaging can also be achieved by exploiting fluorescence signals derived from liposome-based nanostructures, as investigated by Yang et al. [115]. A fluorescence imaging signal in the near-infrared window was reported when lanthanide nanoparticles were covered by lipids and administered in different in vivo areas, as brown adipose tissue or lymph nodes. An interesting aspect of this nanoconstruct was its natural accumulation in brown adipose tissue despite the absence of any targeting molecules, which offered an opportunity for an alternative and sensitive method of imaging in the field of metabolic disorders or micro tumor detection.

Other examples of in vivo studies which involved nanoparticles of a different nature encased in liposome are listed in Table 2. They have the aim of performing gene editing [38], as well as metastasis growth inhibition [116], or other purposes with the same driving design strategy as the studies reported above.

### 3.2. Cell-Derived Extracellular Vesicle-Coated NPs in In Vivo Studies

As already described in Section 2.2, EVs can be exploited as a coating for solid-state nanoparticles and were explored in the literature to improve the efficacy of the nanoconstruct as an anticancer drug in various ways. Despite the greater compatibility and specificity toward cancerous cells that can be achieved by EV coating, research is still in its early stage because EV characterization has only become possible in recent years with the most modern instrumentation. Moreover, their isolation and manipulation still require complex processes which limit their use [117], despite the large availability of raw material. Clinical studies are not yet present in the literature for this specific kind of nanoconstruct; however, some in vitro studies were sufficiently promising to allow their continuation in in vivo studies. In this section, we review the works which were able to reach this stage of research.

A clear example of this is represented by the MOFs embedded in the tumor-derived exosomes already mentioned in Section 2.2 [71]. MOF nanoparticles were loaded with proteins and sealed by means of the exosomal membrane. In this work, the authors proved that the loading of a therapeutical active protein was efficient in bringing it up to the tumor site, resulting in tumor growth inhibition and a great increase in the therapeutic efficacy.

Brain-targeted exosomes were also combined with gold nanoparticles to obtain a device that could overcome the blood–brain barrier and treat brain cancer. In vivo studies revealed that an accumulation in a mice brain could be observed by bioluminescence after the NPs’ intravenous injection [118].

The hybrid gold-iron oxide nanoparticles covered by the cell-derived EVs already cited in Section 2.2 showed promising results in in vivo studies [69]. Biodistribution studies revealed cancer-specific accumulation after intravenous injection into mice. Moreover, magnetic resonance imaging in mice was also demonstrated to be possible with these kinds of NPs, revealing their potential as a theranostic device.

Another theranostic device that was tested in vivo was the one presented in the work of Silva et al. [119]. As already reported, this work focuses on the fabrication of a nanoconstruct that embeds a photosensitizing agent and a magnetic particle into exosomes, exploiting the communicative role of these EVs to efficiently deliver to the tumor target site a theranostic device. A murine model was exploited to prove the efficacy of photodynamic therapy, after which, in the presence of the theranomes subject of the work, a reduction in the growth rate of the tumor was found.

Iron oxide nanoparticles with superparamagnetic properties were analyzed when coupled with exosomes as platforms that can be magnetically manipulated to be separated by blood. These systems were also proved to target the cancer site in vivo, resulting in the inhibition of the tumor growth [120].

Porous silicon nanoparticles were also used as nanocarriers for doxorubicin, a common anticancer drug. An EV shielding was exploited to cover them, resulting in a system that presented enhanced tumor accumulation. In vivo studies showed that the tumor-derived exosomes were able to carry doxorubicin more efficiently to the site of interest, also resulting in higher tumor penetration and enhanced uptake by cancer cells [121].

Again, doxorubicin was exploited to prove the efficacy of a system aimed at tumor drug delivery for EV-coated polymeric NPs. In this case, as already cited in Section 2.2 for the in vitro tests, the in vivo experiments showed an increased tumor accumulation and tumor growth inhibition in mice, proving the efficacy of the therapy [76].

Silica nanoparticles were also exploited in this field. In the work of Wu et al., silica nanoparticles were loaded with a sonosensitizer (indocyanine green) for sonodynamic therapy. Then, the drug-loaded solid nanoparticles were coated with macrophage-derived exosomes able to penetrate the blood–brain barrier for glioblastoma treatment. The aim was confirmed in vivo, where the NPs were able to penetrate the barrier and reduce tumor growth when coupled with ultrasound [122].

**Table 2:** In vivo studies on solid-state nanoparticles enclosed in lipidic-based shell or extracellular vesicles. Green background color refers to artificial liposomes and pink refers to extracellular vesicles. To the authors knowledge, no data are reported in the literature referred to engineered, hybrid or artificial extracellular vesicles.

NPs	Coverage-Liposome	Cargo	Functionalization	Purpose
Au NPs	Lipidic shell (DOPE)	GF2.5-RhB/DNA	Folic acid	Targeted gene delivery [42]
Au NPs	Lipidic monolayer (HPC liposome)	-	-	Improvements in nanoparticle–membrane interactions understanding [43]
Au NPs	Liposome (DOPE, soybean phospholipid, cholesterol, mPEG_2000_-DSPE, and Mal-PEG_2000_-DSPE, DXMS)	Dexamethasone and TGFβ1 siRNA	PEG and α8 integrin antibodies	Therapies for glomerular diseases [123]
Au NPs	Liposome (2 layers) (DSPC, DSPE-PEG, DSPE-PEG-NH_2_, cholesterol)	-	Radiolabeling ^64^Cu	Photothermal therapy of breast cancer [109]
Au NPs, graphene Qdots	Liposome (DSPC, cholesterol)	Doxorubicin	Folic acid ligands	Phototriggered chemotherapy for breast cancer [110]
Au NPs	Lipidic bilayer shell (Lipoid E80)		Au nanorods	Raman-scattering tags for bioimaging and diagnosis applications [41]
Ruthenium(II) polypyridyl complexes	Thermosensitive liposome (DPPC, DSPC, DSPE-PEG_2000_)		Au nanorods	Antitumor activity [112]
Magnetic ZnFe_2_O_4_ NPs	Magneto liposome (soy lecithin, cholesterol, cetyl trimethylammonium bromide)	Imatinib	Hyaluronic acid	Targeted/controlled drug release for cancer therapy [124]
Magnetite NPs	Liposome (egg phosphatidylcholine, phosphatidylinositol)	-	-	MRI contrast agent for enhancement in renal carcinoma tissue [113]
Lanthanide NPs	Liposome (DSPE-PEG_2000_, Cholesterol, DPPC)	-	-	Fluorescence imaging diagnoses [115]
Zn_1.1_Ga_1.8_Ge_0.1_O_4_:Cr^3+^ persistent luminescence NPs	Liposome (DPPC, DSPC, DOPC, cholesterol)	Paclitaxel	-	Luminescence imaging guided chemo-therapy [52]
ZnO NPs	Lipidic shell (DOPA, DSPE-PEG_2000_, cholesterol, DOPC)	6-mercaptopurine	-	Lymphatic-targeted drug carriers [54]
Zn-doped Fe3O4 NPs	Liposome (DSPE-PEG_2000_maleimide)	-	Tumor-penetrating peptide (RGERPPR)	Contrast agent for diagnostic imaging of brain glioma remains [114]
Mesoporous silica	Liposome (DOTAP, DSPE-PEG_2000_, DOPE, cholesterol)	CRISPR components	-	Gene editing in mice brain [38]
QD containing silica NPs	Paramagnetic lipid shell (DSPE-PEG_2000_, Gd-DTPA-DSA)	-	-	Improvements for drug delivery, gene therapy, and molecular imaging based on silica NPs [39]
Bufalin and Fe_3_O_4_	Liposome (DDPC, cholesterol-PEG_2000_, cholesterol)	-	-	Inhibit lymphatic metastasis in breast cancer [116]
QDots	Liposome (DSPC, DOTAP, DSPE-PEG_2000_)	-	-	Increased tumor uptake of fluorescently stable QDs [125]
pH responsive poly-β-amino ester	Phospholipid bilayer shell (DOPC, DOTAP, DSPE-PEG_2000_)	mRNA		Potential utility for mRNA vaccine formulations [58]
CaCO_2_	Lipidic shell (DOPA, DPPC, cholesterol, DSPE-PEG)	Doxorubicin and Oxa(IV)	-	pH-responsive drug delivery system [126]
Calcium/phosphate NPs	Lipidic bilayer (DOPA, DSPE-PEG_2000_)	siRNA	-	Inhibiting metastatic tumor growth [127]
**NPs**	**Coverage-Exosome**	**Cargo**	**Functionalization**	**Purpose**
MOFs NPs	Tumor-derived EVs	Proteins	-	Enhance protein therapy efficacy and tumor targeting [71]
Au NPs	Transformed cell culture-derived exosomes	-	-	Brain cancer treatment [118]
Au-iron oxide NPs	Tumor-derived EVs	microRNA	-	Photodynamic, MRI and gene therapy [69]
Iron oxide NPs	Macrophage-derived EVs	m-THPC(photosensitizer)	-	Photodynamic therapy and MRI [119]
Iron oxide NPs	Blood cell-derived exosomes	Doxorubicin	-	Drug delivery and magnetic manipulation [120]
Porous silicon NPs	Tumor-derived exosomes	Doxorubicin	-	Drug delivery [121]
Silica NPs	Macrophage-derived exosomes	Indocyanine green	-	Sonodynamic therapy [122]
PEOz-PLA NPs	Urinary exosomes	Doxorubicin	-	Drug delivery [76]

In conclusion, the literature has not yet explored, in a major way, the possibilities that are opened by the exploitation of cell-derived EVs to obtain biomimetic, tumor-specific theranostic NPs. The reason may reside in the high specificity of the treatment which can be achieved mainly when the EVs are derived directly from the cell type that is also the target of the therapy. Still, some very specific cases of study revealed that this field is extremely promising, mainly for cancer treatment.

### 3.3. Engineered, Hybrid and Artificial Extracellular Vesicles

Self-assembled liposomes and the possibility to engineer them represents the first step toward a biocompatible and biomimetic method to deliver nanoparticles both in vitro and in vivo for biomedical and antitumoral propositions. Despite the multifunctional flexibility of liposomes, improvements in biomimetic properties must be investigated to design intelligent nanoagents. The creation of hybrid shells composed of artificial liposomes and extracellular vesicles to avoid the immune system [128] represents a viable option. Nevertheless, the synthesis of such composites, and the relative application both in vitro and in vivo, constitutes a challenge for research. This is reflected by the lack of published studies in the biomedical field reporting the results obtained in vivo employing hybrid nanoconstructs loaded with nanoparticles.

During the last decade, research studies on tumor therapies have focused their interest on an alternative strategy: engineered extracellular vesicles. This area, as with the hybrid composites, is still in its twilight zone, as also shown in Figure 3. Few encouraging results are reported for in vitro studies of engineered EVs loaded with NPs, as highlighted in Section 2.3, but the literature in the state of the art for in vivo studies is still scant. One of the few examples is the work of Jia et al. [90]. They evaluated both the diagnostic and therapeutic effects in vivo using SPIONs and curcumin encapsulated in exosomes, which were further engineered with RGE-peptide for targeting. This complex nanoconstruct revealed good in vivo stability and biocompatibility, a strong glioma-targeting ability, an enhanced imaging signal and a strong inhibition of tumor growth. Other exotic mixtures of engineered exosomes and nanoparticles can be found in the literature, as shown in the study of Kwon et al. [92], which was already mentioned in Section 2.3. The authors proposed magnetic nanoparticles not as a cargo, but as a functionalizing agent bound to the surface of doxorubicin-loaded exosomes to exploit the effects of hyperthermia. The addition of folic acid ligands as targeting agents on the exosome surface ensured the absence of damage on healthy tissues in a xenograft mouse model, and the whole nanoconstruct demonstrated an in vivo growth-inhibiting effect on colorectal cancer thanks to the dual, i.e., chemotherapy and hypothermia, strategy.

In the forthcoming years, groundbreaking improvements are expected in this nanomedicine research area, translating the fascinating results of in vitro research to in vivo and 3D models, and opening new strategies to fight cancer.

## 4. 3D Cultures Studies and Perspectives

**Figure 4:**
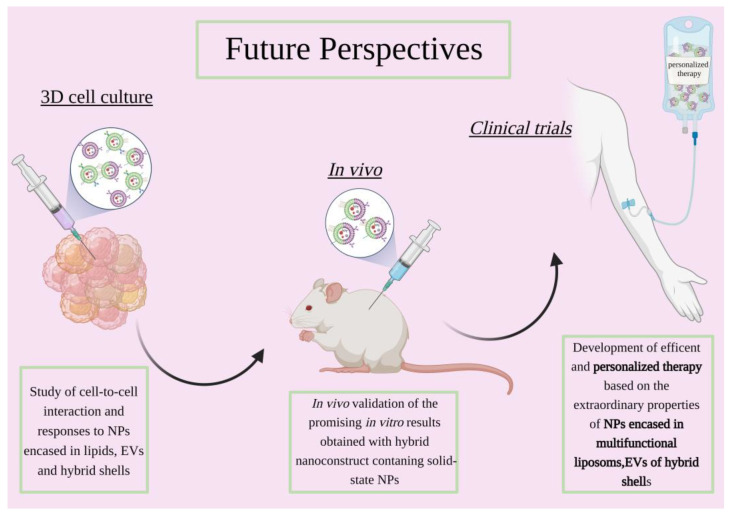
Scheme showing the future perspective of anticancer research involving solid-state nanoparticles enclosed in lipidic-based shells, extracellular vesicles or engineered hybrid vesicles. Created with BioRender.com, accessed on 26 October 2022.

The selection process for novel therapeutic approaches is complex and requires adequate testing for it to succeed during clinical trials [129]. For this purpose, 2D in vitro models have been widely favoured due to their high feasibility, versatility and cost-effectiveness [130,131]. However, two-dimensional models have proven, over time, to be lacking in the complexity and accuracy required to aptly mimic the tumor microenvironment, thus leading to unreliable results in terms of drug screening [130,132]. Early in vitro models were flanked by the in vivo ones that had represented, for many years, the most valuable platform for drug screening. However, in more recent times, the emergence of 3D in vitro models has allowed cancer research to overcome some of the drawbacks linked to the use of murine models. Notwithstanding the ethical concerns that have also arisen, in vivo models fail to mimic the complexity of the human body as they do not recapitulate the heterogenous tumor microenvironment and use platforms which lack an immune system and still differ from human in vivo activity in terms of metabolism and excretion. To overcome said limitations, 3D in vitro models have been presented as an alternative to better recapitulate the various tumor features found in vivo [133], as schematized in Figure 4. Most importantly, key aspects such as cell–cell interactions and cell–ECM interactions can be reproduced, allowing for more precise human cancer pathobiology studies [134,135]. Still, 3D cultures are relatively new approaches which are in constant evolution and require novel skills which make them not immediately feasible for novel constructs. This aspect, together with the novelty of the field analysed in this review, i.e., the phospholipidic-membrane coating of solid-state nanoparticles, limits the availability of the research literature that employs these two biomedicine branches together. Still, 3D in vitro studies pave the way for a lot of possibilities which could soon be exploited to validate the nanoconstructs prior to their test in vivo. Therefore, some of the recent findings in the field of liposome-based nanomedical devices will be reviewed as well, in order to provide a panorama of the methodologies that could be easily exported to coat solid-state nanoparticles with lipid, EVs and more engineered membranes.

In fact, 3D cancer models have proven to be useful when evaluating the efficacy and potential of liposomes for oncological therapy. Specifically, cancer spheroids have been employed as tools to test various active-targeting approaches. Tavares Luiz et al. modified liposomes with folic acid (FA) and loaded them with curcumin in order to treat breast cancer [136]. For this purpose, MCF-7 spheroids were obtained using the liquid overlay technique and were used to compare the viability and cellular uptake induced by curcumin alone (CCM), liposomes loaded with curcumin (LIP-CCM) and FA-modified liposomes loaded with curcumin (FA-LIP-CCM). These results proved not only the limitations of two-dimensional systems for drug testing, but also the importance of folic acid functionalization for active targeting [136]. Similarly, T7 peptide was used by Riaz et al. for functionalizing liposomes for the active targeting of the transferrin receptor and loaded with quercetin to act as a chemotherapeutic agent against lung cancer 3D models [137]. The results not only showed the improvement of tumor penetration thanks to the T7 functionalization, but also highlighted the T7-QR-LIP formulation as the most effective in preventing tumor growth [137]. Liang et al. developed 3D intestinal spheroids using the Caco-2 cell line to test Cyanidin-3-O-Glucoside (C3G), another anticancer flavonoid, loaded liposomes [138]. Compared to what was observed in the before-mentioned studies, the IC_50_ values for the 2D model were significantly lower than those obtained with the 3D model (0.18 mg/mL compared to 0.25 mg/mL), showing a much greater reliability of the more complex model [138]. Finally, Hartwig et al. investigated the treatment of neuroblastoma with an injectable liposomal Cu(DDC)_2_ formulation with anticancer properties [129]. The 3D spheroids were generated using the liquid overlay method with the LS neuroblastoma cell line and the hSkMC and hDFa primary cells [129]. The cytotoxic effect obtained in the 2D models was compared with that of the 3D models for PEGylated and non-PEGylated Cu(DDC)_2_ liposomes 72 h after treatment. The two formulations did not show a significant difference in terms of efficacy, while a clear difference was seen when comparing the two models, as the EC_50_ values obtained after treatment of the 3D spheroids were much higher than those seen for the 2D. Furthermore, the study compared the activity of the liposomal formulation with that of the free Cu^2+^ and free Cu(DDC)_2,_ proving not only that the complex had anticancer properties but also that this activity was maximized in combination with a lipid nanocarrier [129].

In summary, 3D cultures have already demonstrated their effectiveness in better predicting outcomes in more complex biological systems, and research is also taking its first steps toward the application of this paradigm in the engineering of lipid-coated nanoparticles.

## 5. Clinical Studies and Current Limitations of Nanomedicine Applications

Advances in nanomedicine have improved the efficacy of current cancer treatment regimens by overcoming the conventional systemic administrations of bioactive agents.

However, that big success is restricted to only a part of the nanostructures validated in the past years in vitro as in vivo, against solid and hematopoietic tumors. To date, only liposomes and EVs are even under clinical trial for cancer treatments, but the promising in vitro and in vivo results obtained with lipid-coated solid-state nanoparticles indicate that clinical studies can also start heading in this direction.

Comparative clinical studies have shown that nanopharmaceuticals are effective in modifying the pharmacokinetics and biodistribution of the bioactive agent, with the advantage of reducing their systemic exposure and toxicity. As a result, the developed nanomedicines led to an increased overall survival of patients and improved clinical outcomes [139,140,141].

Although the clinical results obtained with the use of nanomedicine have been promising, there is still a long way to go to treat advanced and metastatic cancers. With the aim of developing more effective anticancer nanopharmaceutics, further and more in-depth investigations are, therefore, needed.

Despite the significant progress made on the biodistribution, one of the limitations of the current state that needs further study work is that often only a small portion (mostly < 1%) of systemically injected NPs reach the tumor site [142].

Generally, many complex factors must be considered in the design of a nanoformulation capable of effectively reaching the tumor, accumulating and persisting intratumorally. The most important factors are NP size, surface feature, stability and their interactions with the tumor microenvironment. For example, NPs with a hydrodynamic dimension > 100 nm are promptly absorbed by the endothelial reticulum system, while NPs with a hydrodynamic dimension < 10 nm are rapidly eliminated from the bloodstream through the glomerular filtration of the kidneys [143,144]. Thus, a hydrodynamic size range of NPs between 10 and 100 nm is required to lessen their clearance and extend their blood half-life. In addition, within this optimum size range, smaller NPs manage to penetrate the tumor more deeply in the core and to distribute more homogeneously within the tumoral interstitium as they are subjected to a significantly lower degree of diffusional hindrance [145,146].

Another factor to consider is the protein corona formed on the surface of NPs that is responsible for the fast clearance of NPs by the mononuclear phagocytic system owing to the possibility to affect the release profile of encapsulated drugs, their targeting capability and therapeutic efficacy, while reducing the circulation time of NPs [147,148]. One way to minimize nonspecific protein adsorption and enhance the blood circulation time of NPs is to bring some changes on their surface through different approaches, such as coating with PEG, polysorbate 80, dysopsonin proteins (e.g., clusterin and albumin), self-markers (e.g., CD47 peptides) and ‘camouflaging’ with a membrane of leukocytes, erythrocytes or thrombocytes [27,149].

Concerning drug delivery systems, to date, the FDA approved exclusively cancer nanomedicines based on liposomes characterized by a higher drug loading efficiency, greater systemic stability, longer circulation time with respect to standard chemotherapy and the capability of encapsulating both hydrophilic and hydrophobic molecules [150,151,152,153,154,155].

Another category of anticancer nanopharmaceuticals that would be worth exploring with further studies is polymeric NPs (e.g., BIND-014 and CRLX101) [152,153].

In the context of preclinical research, current in vivo models cannot accurately summarize the enhanced permeability and retention (EPR) effect in patients because tumor features in animal models and patients are obviously not comparable in terms of growth rate, relative size body mass and microenvironment characteristics [154]. The EPR effect in patients is affected by disease stage, tumor histotype, size and heterogeneity, as well as interpatient variability, resulting in it not being as prominent as in animal models [155,156,157]. Therefore, with the aim of reaching successful clinical trials, the study and development of efficient biomarkers and imaging tools to predict the extent of EPR is essential. The identification of specific predictive biomarkers of EPR will enable the preselection of patients based on the extent of EPR, ensuring a high performance for cancer nanomedicines in future clinical translation [157,158,159,160].

Another way to overcome the restrictive passive targeting of tumors via EPR could be the coating of the surface of NPs with specific targeting molecules recognized and bound by receptors well expressed by cancer cells or angiogenic endothelial cells. Active targeting between the ligand and receptor could result in the NP uptake and internalization into tumor cells, enhancing antitumor activity and limiting off-target toxicity [161,162].

Considering the heterogeneity of tumors, coating NPs with several specific molecules that target more than one surface receptor, chosen based on their skill for efficient internalization and endosomal escape, could be advantageous [163]. Moreover, in that setting, a patient tumor genetic sequenome is strongly recommended to select the targets based on their expression.

In the last decade, immunotherapy has had a positive impact on clinical cancer care. Immunotherapeutic drugs, such as checkpoint inhibitors, are being validated and administered in combination with other cancer therapeutics to improve the clinical outcomes of current treatments [164,165]. For example, in 2019, the FDA approved the combined treatment of atezolizumab [antiprogrammed death-ligand 1 (PD-L1)] with nab-PTX for PDL1-positive unresectable locally advanced or metastatic triple-negative breast cancer. In a Phase III trial, that enrolled patients with PD-L1-positive tumors, it was reported that the combination between nab-PTX and atezolizumab increased median progression-free survival from 5 months to 7.5 months (HR 0.62; 95% CI) and overall survival from 15.5 months to 25 months (HR 0.62; 95% CI) for nab-PTX and atezolizumab, respectively [166].

Feasibly combining nanomedicines with immunotherapy will lead to future positive clinical outcomes in the field of cancer therapy.

To date, nanomedicines based on liposomes and EVs have significantly improved the clinical trial outcomes of cancer patients, and, probably, in the near future, active-targeted nanomedicine will play an important role in providing innovative therapeutic approaches thanks to better formulations, reduced systemic exposure and toxicity. Moreover, the combination of multitherapeutic agents or therapeutic regimens such as chemo-, radio-, thermo-, gene and immunotherapy will possibly enhance the clinical efficacy of nanomedicines in the treatment of cancer. In this context, artificial and naturally derived lipidic bilayers exploited to coat solid-state NPs seem a more than bright path to explore in clinical research in the coming years. However, patient preselection based on the extent of EPR, the expression of target receptors and tumor heterogeneity are required for patients to further benefit from the superior therapeutic outcomes of cancer nanomedicines.

## 6. Conclusions and Future Perspectives

The use of phospholipid membranes is already well established, both in the literature and in clinic, to ferry a wide variety of therapeutic molecules. However, its exploitation as a carrier of solid-state nanoparticles is still in the early stages of research. Nonetheless, this branch of biomedicine is very active and is producing an increasing number of papers, reporting promising results. The literature review presented here inquires into the state of the art of phospholipid-coated solid-state nanoparticles, analyzing which works have achieved in vivo validations and whether there are systems that have reached clinical trials. This analysis is also carried out by relating the level of progress achieved by the research into the origin of the lipid membrane and the advantages and disadvantages of differently derived lipids.

Artificial lipid-coated NPs are certainly those that present the greatest amount of the related/dedicated literature, thanks to the previous and extensive exploitation of liposomes in clinics. For the moment, the trend of research is toward the manufacture of devices that substantially mimic the original purpose for which liposomes were introduced in nanomedicine, namely the administration of drugs. Another series of works reports the exploitation of nanoparticles that is already well characterized in the literature, and which intrinsically have interesting therapeutic and imaging potentials. In vitro studies have shown promising results for these nanoconstructs, and a substantial part of the work has also been tested in vivo. However, the high reproducibility of commercially available artificial lipid-based systems is thwarted by their struggle to naturally promote tumor targeting, also limiting their exploitation in clinical trials.

Therefore, the line of research has split into a parallel branch that sees naturally derived EVs as a coating for solid-state NPs. In most of the articles that can be found in the literature at the time of writing, EVs were derived from tumor cell lines due to their innate selectivity toward the cells they come from. In addition, in this case, there are several works that report good results in vitro; however, the relative novelty of this field, together with the difficulties in the extraction and storage of EVs, still limits the exploitation of EV-coated solid NPs in vivo and, in turn, also in clinical tests.

Engineered and hybrid liposomes/EV-coated NPs represent the last frontier toward which research is heading, in trying to understand how to solve all the aforementioned limitations. Despite the promising results presented in vitro, this field is still new, and it will take time to bring them to in vivo studies and possibly 3D cultures and clinical applications.

In conclusion, both artificial and naturally derived phospholipidic coatings appear to be the main avenue for the exploitation of solid nanoparticles in nanomedicine. However, the research is still in its infancy and, therefore, many limitations have yet to be overcome.

## Data Availability

Not applicable.
